# Amelioration of hypercalcemia by cinacalcet treatment in a subject with relapsing acquired hypocalciuric hypercalcemia: A case report

**DOI:** 10.1097/MD.0000000000027579

**Published:** 2021-10-22

**Authors:** Junpei Sanada, Shinji Kamei, Masashi Shimoda, Fuminori Tatsumi, Tomohiko Kimura, Atsushi Obata, Kenji Kohara, Shuhei Nakanishi, Kohei Kaku, Tomoatsu Mune, Hideaki Kaneto

**Affiliations:** Department of Diabetes, Endocrinology and Metabolism, Kawasaki Medical School, Kurashiki, Japan.

**Keywords:** acquired hypocalciuric hypercalcemia, case report, cinacalcet, prednisolone

## Abstract

**Rationale:**

Hypocalciuric hypercalcemia is classified as acquired hypocalciuric hypercalcemia (AHH) and familial hypocalciuric hypercalcemia (FHH). While FHH is inherited as an autosomal dominant trait, AHH is one of the rare acquired diseases and is usually treated with prednisolone. Here, we report a case with relapsing AHH which was well controlled with cinacalcet therapy.

**Patient concern:**

A 68-year-old Japanese man was referred to our institution because of hypercalcemia. Despite such hypercalcemia, he was almost asymptomatic.

**Diagnostics:**

We diagnosed him as AHH due to the following reason. First, the ratio of calcium (Ca)/creatinine clearance was very low which met the criteria. Second, there was no overt family history of hypercalcemia. Third, his serum Ca level was within the normal range 3 years before. Fourth, despite hypercalcemia, he was almost asymptomatic and had no evidence of primary hyperparathyroidism.

**Interventions:**

Although it is known that steroid therapy is useful for AHH, optimal treatment remains unknown and cinacalcet therapy is very much limited for the treatment of AHH. In this subject, we introduced cinacalcet therapy for the treatment of relapsing AHH.

**Outcomes:**

Serum Ca and parathyroid hormone levels were normalized after such therapy with cinacalcet.

**Conclusions:**

We should bear in mind that cinacalcet treatment is effective for the treatment of relapsing AHH.

## 1 Introduction

Hypocalciuric hypercalcemia is brought out in subjects with acquired hypocalciuric hypercalcemia (AHH) or familial hypocalciuric hypercalcemia (FHH). FHH is induced by a gene mutation in the Ca^2+^-sensing receptor (CaSR) and is inherited as an autosomal dominant trait.^[1–3]^ In contrast, AHH is one of the rare acquired diseases and is usually treated with prednisolone. Another possible therapeutic option is calcimimetics such as cinacalcet hydrochloride. Cinacalcet is often used for the treatment of secondary hyperparathyroidism in dialysis subjects or hypercalcemia due to parathyroid carcinoma.^[4]^ However, such cinacalcet treatment is much more limited for the treatment of AHH. To the best of our knowledge, there are only a few reports showing the effectiveness of cinacalcet in a subject with AHH.^[5]^

Here, we show a case of relapsing AHH which was well controlled by cinacalcet treatment.

## 2 Case presentation

A 68-year-old Japanese man was referred to our institution because of hypercalcemia. At that time, calcium (Ca) level was elevated to 13.1 mg/dL, although 3 years before it was within the normal range (9.3 mg/dL). He had no family history of consanguineous marriage and hypercalcemia. Indeed, serum Ca levels in 2 brothers were within the normal range. To further examine the cause of hypercalcemia, he was hospitalized in our institution.

His height and body weight were 157 cm and 52.1 kg. As shown in Table 1, serum Ca level was markedly elevated to 14.1 mg/dL and serum inorganic phosphorus level was decreased to 2.2 mg/dL. Urinary Ca excretion was 180 mg/24 h, creatinine clearance was 164 mL/min and the ratio of Ca/creatinine (Cre) clearance (Cca/Ccr) was 0.004. In addition, we measured this value on the other 2 different days and similar results were obtained (Cca/Ccr, 0.006 and 0.005). Cca/Ccr ratio met the criteria (<0.01)^[6]^ on 3 different days. The intact parathyroid hormone (PTH) level was slightly elevated to 58.0 pg/mL. PTH-related protein (PTHrP) was not detectable. There were no abnormalities in an electrocardiogram, a neck echogram, and whole-body scintigraphy with ^9m^Tc methoxyisobutylisonitrile. Thyroid function was normal and there was no evidence of other hormonal disorders and sarcoidosis.

**Table 1:** Clinical parameters on admission in this subject.

Parameter	Results	Reference	Parameters	Results	Reference
WBC	4840/μL	3300–8600	ACTH	71.3 pg/mL	7.2–63.3
Hemoglobin	14.4 g/dL	13.7–16.8	Cortisol	18.4 μg/dL	6.24–18.0
Platelet	21.9 × 10^4^/μL	15.8–34.8	DHEA-S	73.0 μg/dL	24–244
AST	25 U/L	13–30	TSH	2.49 μU/mL	0.35–4.94
ALT	16 U/L	10–42	FT3	3.38 pg/mL	1.71–3.71
LDH	170 U/L	124–222	FT4	1.02 ng/dL	0.70–1.48
ALP	319 U/L	106–322	CEA	2.9 ng/mL	≤5.0
T-bilirubin	0.7 mg/dL	0.4–1.5	CA19–9	≤5 U/mL	≤37.0
Albumin	4.4 g/dL	4.1–5.1	PSA	3.52 ng/mL	<4.0
Creatinine	0.88 mg/dL	0.65–1.07	SCC	1.4 ng/mL	≤1.5
BUN	16 mg/dL	8–20	sIL-2R	249 U/mL	145–519
UA	6.6 mg/dL	3.7–7.8	NTX	18.9 nmolBCE/L	7.5–16.5
CRP	0.04 mg/dL	≤0.14	TRACP-5b	331 mU/dL	170–590
Sodium	143 mEq/L	138–145	BAP	17.3 μg/L	3.7–20.9
Potassium	4.7 mEq/L	3.6–4.8	ucOC	6.37 ng/mL	<4.5
Chlorine	106 mEq/L	101–108			
Calcium	14.1 mg/dL	8.5–10.2	(Urine)		
Phosphorus	2.2 mg/dL	2.4–4.3	Sodium	73 mEq/L	40–90
Intact PTH	58 pg/mL	10–65	Potassium	17 mEq/L	20–60
PTHrP	≤1.0 pmol/L	≤1.0	Chlorine	68 mEq/L	40–120
1,25 (OH)_2_ VitD_3_	79.8 pg/mL	20.0–60.0	Ccr	93.2 mL/min	90–120
osmolality	295 mOsm/kg	276–292	Osmolality	297 mOsm/kg	50–1300
BNP	15.1 pg/mL	≤18.4	FECa	0.004	0.02–0.04
ACE	14.6 U/L	8.3–21.4	%TRP	78%	81–90

Taken together, we excluded various causes of hypercalcemia in this subject. First, we excluded the possibility of primary hyperparathyroidism and hyperthyroidism based on blood test results. Second, we excluded the possibility of sarcoidosis because ACE was negative and there was no bilateral hilar lymph node enlargement in chest CT. Third, we excluded the possibility of malignancy because PTHrP was negative and there were no findings indicating malignancy in the chest and abdominal CT. Fourth, while it is known that tuberculosis and granulomatous diseases can be accompanied by hypercalcemia, we excluded the possibility of such disorders because there were no findings indicating such disorders in the chest and abdominal CT. Fifth, this subject did not take any supplemental food and/or any medicine which can bring about hypercalcemia such as thiazide diuretic. Finally, although it is known that hypercalcemia can be induced by long-term bed rest, this subject did not experience long-term bed rest and thus we think that there was no possibility that hypercalcemia was induced by such reason in this subject.

**Figure 1:**
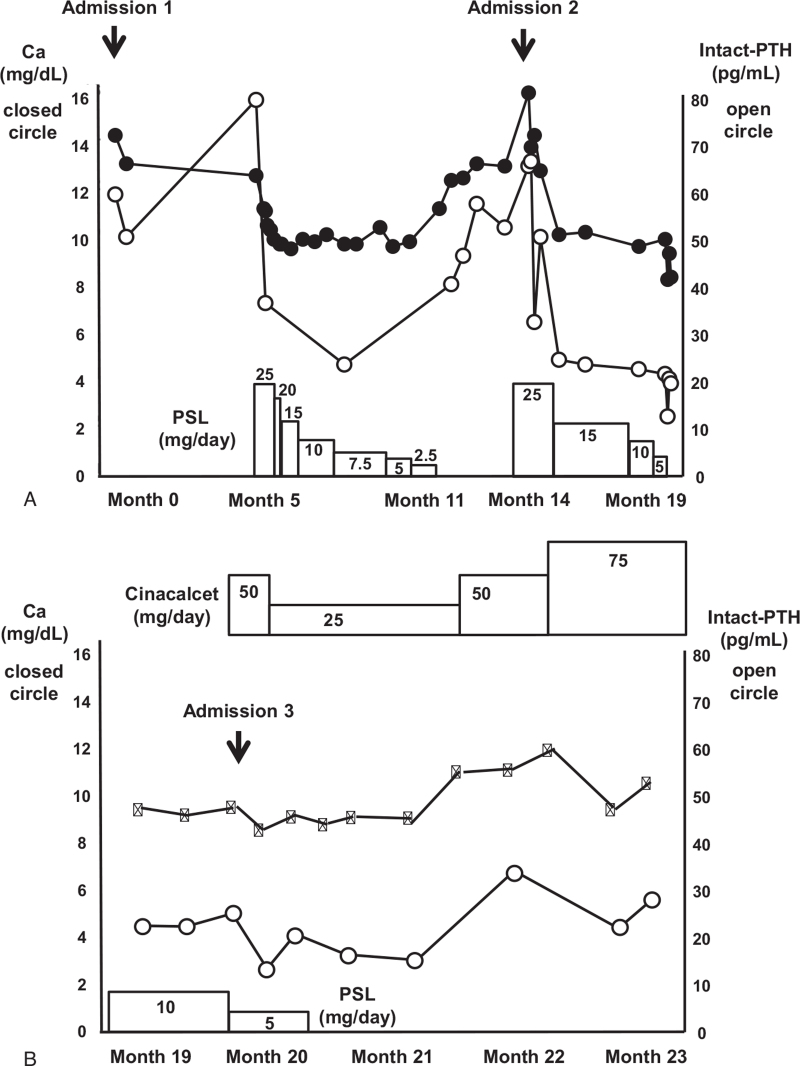
(A) Effects of PSL treatment and (B) effects of cinacalcet treatment on serum Ca and intact PTH levels in a subject with AHH. Closed circle: serum Ca levels; open circle: serum intact PTH levels. AHH = acquired hypocalciuric hypercalcemia, PSL = prednisolone.

Given these findings and the following reasons, we diagnosed this subject as AHH. First, unlike typical FHH, there was no overt familial history of hypercalcemia. Second, his serum Ca level was within the normal range 3 years before this admission, which was compatible with the acquired phenomenon. Finally, despite hypercalcemia, he was almost asymptomatic and had no evidence of primary hyperparathyroidism. Furthermore, throughout the clinical course, the synchronous pattern between serum Ca and PTH levels was observed (Fig. 1). After the diagnosis, we started 25 mg/day of prednisolone therapy. After then, his serum Ca level was rapidly decreased to within normal range (9.5 mg/dL), and PTH level was also decreased to within normal range (28 pg/mL) synchronously to serum Ca level. After tapering prednisolone to 20 mg/day, he was discharged (Fig. 1A). Prednisolone therapy was performed for 7 months with tapering of prednisolone to 5 mg/day. However, hypercalcemia relapsed several months later. Therefore, he was hospitalized again and we re-started 25 mg/day of prednisolone and gradually decreased. After then, serum Ca levels were within the normal range (Fig. 1A).

Since cinacalcet became available in Japan under health insurance for primary hyperparathyroidism and other conditions, we started cinacalcet treatment together with prednisolone. He was treated with cinacalcet at a starting dose of 50 mg/day. After then, the Ca level was decreased to 8.8 mg/dL 4 days later, we reduced the cinacalcet dose to 25 mg/day. (Fig. 1B). PTH level also became within normal range. After discharge, serum Ca level was maintained with 25 mg/day of cinacalcet combined with 5 mg/day of prednisolone. Therefore, we tapered off prednisolone and continued monotherapy of cinacalcet. Since serum Ca level was gradually increased up to 11.6 mg/dL, we increased the dose of cinacalcet, from 25 to 75 mg/day. After then, a good response was observed; serum Ca level was decreased to 10.3 mg/dL with monotherapy of cinacalcet. Finally, relapsing AHH even after steroid therapy was well controlled with cinacalcet monotherapy.

## 3 Discussion and conclusions

Here, we showed that hypercalcemia was ameliorated by cinacalcet treatment in a subject with relapsing AHH. We diagnosed this subject as AHH due to the following reason. The ratio of Cca/Ccr was as low as 0.004, which met the criteria (<0.01).^[6]^ Unlike typical FHH,^[1–3]^ there was no overt family history of hypercalcemia. His serum Ca level was within the normal range 3 years before. Despite hypercalcemia, he was almost asymptomatic and had no evidence of primary hyperparathyroidism.

Regarding the mechanism of action of cinacalcet, it is known that cinacalcet acts on calcium receptors and reduces PTH secretion from the parathyroid gland. In addition, generally we start cinacalcet at 50 mg/day and adjust its dose while checking serum Ca level and its possible side effects. Needless to say, however, there are no clear criteria about an initial dose in subjects with relapsing AHH as observed in this subject. Digestive symptoms such as nausea are observed as side effects of cinacalcet. It has been reported that CaSR is expressed in the Auerbach plexus and may be involved in intestinal peristalsis.^[7,8]^

In this subject, however, there was no special side effect with which we had to consider discontinuing its medication. In addition, we should be careful when using cinacalcet in elderly subjects with liver dysfunction, active gastric ulcer, or a convulsive seizure. This subject had a past history of gastric ulcers, but there was no special problem because we started using cinacalcet about 2 years after the recovery of gastric ulcer.

Cinacalcet is often used for the treatment of secondary hyperparathyroidism in dialysis patients and hypercalcemia due to parathyroid carcinoma.^[4]^ To the best of our knowledge, however, there are only a few reports showing the effectiveness of cinacalcet in a subject with AHH.^[5]^ Interestingly, our patient had some features which were different from the previously reported case.^[5]^ First, our patient had no other autoimmune diseases. Because our patient had no evidence of systemic autoimmune diseases such as rheumatoid arthritis, systemic lupus erythematodes, and autoimmune thyroid disease, we assumed that immuno-target was restricted to the CaSR in this subject due to some unknown reason. Second, serum Ca level was markedly higher compared to the reported case.^[5]^ It is known that PTH functions in the parathyroid glands, the kidney, and/or the bone. Indeed, CaSR expression is observed in osteoblasts or osteoclasts, suggesting that it can enhance the Ca efflux from the bone. Therefore, although speculative, we think the possibility that PTH more strongly facilitated Ca efflux from the bone in this subject.

In general, it is well known that steroid therapy is very useful to reduce autoimmunity in various tissues but that such therapy for a long period of time is usually accompanied by various side effects such as susceptibility to infection, impaired glucose tolerance,^[9]^ osteoporosis,^[10]^ and stomach or duodenal ulcer. In this subject as well, when we started the treatment with cinacalcet, we stopped steroid therapy to avoid the above-mentioned side effects of long-term steroid therapy. After changing prednisolone to cinacalcet, serum Ca level was slightly increased in this subject. However, we think that the possible side effects of long-term steroid therapy would far outweigh its beneficial effects on this subject.

In conclusion, cinacalcet treatment together with conventional prednisolone therapy successfully normalized serum Ca and PTH levels without any side effect in a subject with relapsing AHH even after steroid therapy. We should bear in mind that cinacalcet treatment is effective for the treatment of relapsing AHH.

## Author contributions

JS, SK, and HK researched data and/or wrote the manuscript and MS, FT, TK, AO, KK, SN, KK, and TM contributed to the discussion.

**Data curation:** Junpei Sanada.

**Investigation:** Junpei Sanada.

**Supervision:** Tomoatsu Mune.

**Validation:** Masashi Shimoda, Fuminori Tatsumi, Tomohiko Kimura, Atsushi Obata, Kenji Kohara, Shuhei Nakanishi, Kohei Kaku, Tomoatsu Mune.

**Writing – original draft:** Junpei Sanada, Shinji Kamei.

**Writing – review & editing:** Hideaki Kaneto.
